# Supplementation of Vitamin D in the Postdelivery Period of Women with Previous Gestational Diabetes Mellitus: Systematic Review and Meta-Analysis of Randomized Trials

**DOI:** 10.1055/s-0041-1734000

**Published:** 2021-10-20

**Authors:** Meline Rossetto Kron-Rodrigues, Marilza Vieira Cunha Rudge, Silvana Andrea Molina Lima

**Affiliations:** 1 Universidade Guarulhos, Programa de Pós-Graduação em Enfermagem (Stricto Sensu), Guarulhos, SP, Brazil; 2 Departamento de Ginecologia e Obstetrícia, Universidade Estadual Paulista “Júlio de Mesquita Filho,” Botucatu, SP, Brazil; 3 Departamento de Enfermagem, Universidade Estadual Paulista “Júlio de Mesquita Filho”, Botucatu, SP, Brazil

**Keywords:** gestational diabetes, vitamin D, meta-analysis, postpartum period, systematic review, diabetes gestacional, vitamina D, metanálise, período pós-parto, revisão sistemática

## Abstract

**Objective** To evaluate the effects of vitamin D supplementation in the postpartum period of women with previous gestational diabetes mellitus (GDM).

**Methods** Randomized clinical trials of pregnant women with GDM of any chronological, gestational age and parity, with no history of previous disease who received vitamin D supplementation in the prenatal and/or postpartum period and were evaluated in the postpartum period were included. The PubMed, EMBASE, Cochrane, and LILACS databases were consulted until July 2019. Serum vitamin D concentration (25-hydroxyvitamin D in nmol/L), fasting blood glucose, glycated hemoglobin, serum calcium concentration, homeostatic model assessment of insulin resistance (HOMA-IR), quantitative insulin sensitivity check index (QUICKI), parathyroid hormone (PTH) and body mass index (BMI) were evaluated. Similar results in at least two trials were plotted using the RevMan 5; Cochrane Collaboration, Oxford, Reino Unido. The quality of the evidence was generated according to the classification, development, and evaluation of the classification of the recommendations.

**Results** Four studies were included in the present review (200 women). The findings indicate that there is no difference in the postpartum period in women diagnosed with previous GDM who received vitamin D supplementation in the prenatal and/or in the postpartum period, showing only that there was a significant increase in the concentration of vitamin D (relative risk [RR]: 1.85; 95% confidence interval [CI]: 1.02–2.68).

**Conclusion** This increase in the concentration of vitamin D should be interpreted with caution, since the assessment of the quality of the evidence was very low. For the other analyzed outcomes, there was no significance between the intervention and control groups, and the outcomes, when analyzed in their strength of evidence, were considered very low and low in their evaluation.

## Resumo

**Objetivo** Avaliar os efeitos da suplementação de vitamina D no pós-parto de mulheres com diabetes mellitus gestacional (DGM) anterior.

**Métodos** Foram incluídos ensaios clínicos randomizados com gestantes com GDM de qualquer idade cronológica, gestacional e paridade, sem história de doença prévia, que receberam suplementação de vitamina D no pré-natal e/ou no pós-parto e foram avaliadas no pós-parto. As bases de dados consultadas foram PubMed, EMBASE, Cochrane e LILACS, até julho de 2019. Foram avaliados concentração sérica da vitamina D (25-hidroxivitamina D em nmol/L), glicemia de jejum, hemoglobina glicada, concentração sérica de cálcio, modelo de avaliação da homeostase de resistência à insulina (HOMA-IR, na sigla em inglês), índice qualitativo *de verificação da* sensibilidade *à* insulina (QUICKI, na sigla em inglês), hormônio da paratireoide (PTH) e índice de massa corpórea (IMC). Resultados semelhantes em pelo menos dois ensaios foram plotados no software RevMan 5; Cochrane Collaboration, Oxford, Reino Unido. A qualidade das evidências foi gerada de acordo com a classificação, o desenvolvimento e a avaliação da classificação das recomendações.

**Resultados** Quatro estudos foram incluídos na presente revisão (200 mulheres). Os achados indicam que não há diferença no período pós-parto em mulheres com diagnóstico prévio de DMG que receberam suplementação de vitamina D no período pré-natal e/ou pós-parto, mostrando apenas que houve um aumento significativo na concentração de vitamina D (risco relativo [RR]: 1,85; IC [intervalo de confiança] 95%: 1,02–2,68).

**Conclusão** Este aumento na concentração de vitamina D deve ser interpretado com cautela, uma vez que a avaliação da qualidade das evidências foi muito baixa. Para os demais desfechos analisados, não houve significância entre os grupos intervenção e controle, e os desfechos, quando analisados em sua força da evidência, foram considerados muito baixa e baixa em sua avaliação.

**PROSPERO** CRD42018110729

## Introduction

Diabetes mellitus (DM) is characterized as a heterogeneous group of metabolic disorders that have hyperglycemia in common, resulting from defects in the action and/or in the secretion of insulin. 1 Diabetes mellitus can be classified into four general categories: type 1 diabetes, type 2 diabetes, gestational diabetes mellitus (GDM), and other specific types of diabetes. 2

For many years, GDM has been characterized as any degree of glucose intolerance recognized for the first time during pregnancy, regardless of whether the disease started before pregnancy or even persisted after pregnancy. This definition facilitated a uniform tracking strategy but was limited by imprecision. 2

Currently, there is an epidemic of obesity in the population of childbearing age, which creates the possibility of the pre-existence of type 2 diabetes with underdiagnosis. Thus, GDM is defined as diabetes diagnosed in the 2 ^nd^ or 3 ^rd^ trimester of pregnancy that was not clearly diabetes prior to pregnancy. 3 Gestational DM occurs in 1 to 14% of all pregnancies and is related to increased perinatal morbidity and mortality. In Brazil, ∼ 7% of pregnancies are complicated by gestational hyperglycemia. 1

The type of DM with the highest incidence in pregnancy is GDM, followed by pre-existing type 1 diabetes and type 2 diabetes. Regardless of the type of diabetes, the specific risks of uncontrolled diabetes in pregnancy include miscarriage, fetal abnormalities, pre-eclampsia, fetal death, macrosomia, neonatal hypoglycemia, and neonatal hyperbilirubinemia, among others. In addition, diabetes in pregnancy can increase the risk of obesity and type 2 diabetes in children born to diabetic mothers. 3 4 During the fetal phase, the organs and tissues undergo critical periods of maturation, concomitantly with phases of rapid cell division. 5

Fetal programming is characterized as a process by which a stimulus or insult, when received in the critical period of development, results in permanent repercussions on the structure and functions of the organism. 6 Continuous transformations in the physiological processes of fetal programming can alter patterns of gene expression with influences on functions and phenotypes. 6

It is estimated that nutrients can modify the immune and metabolic programming during sensitive periods of fetal and postnatal development. Among these nutrients, vitamin D stands out, since current observational studies suggest that it is essential for many physiological processes. 7 8 9

With the vitamin definition, vitamin D3 or cholecalciferol can be synthesized by mammals from 7-dehydrocholesterol and through exposure to ultraviolet irradiation. Cholecalciferol or ergocalciferol (vitamin D2) can be obtained from dietary sources. In humans, cholecalciferol and ergocalciferol are sequentially transformed into 25-hydroxyvitamin D3 (25 OH VD), 25-hydroxycholecalciferol, or calcidiol, in the liver, and are subsequently transformed in the kidneys and other tissues into 1,25-dihydroxyvitamin D3 (1, 25 [OH] 2D), 1,25-dihydroxycholecalciferol or calcitriol. 7 8 9

Vitamin D is responsible for maintaining calcium homeostasis and bone formation, including its relationship with the immune system. 10 Vitamin D is known to have immunomodulatory and anti-inflammatory effects. 11 Observational studies have shown a link between vitamin D deficiency and the onset and progression of type 2 diabetes. 12 13

The literature points out that maternal vitamin D deficiency during pregnancy can have negative consequences for maternal and fetal health and is also associated with greater maternal and perinatal risks, such as: higher incidence of preeclampsia, insulin resistance, development of GDM, and increased frequency of cesarean delivery. 7 8 10 11 12 13 14 15

Two recent meta-analyzes that aimed to assess the effects of vitamin D supplementation during the pregnancy of normoglycemic pregnant women on obstetric outcomes and birth variables reported that birthweight and newborn length were significantly higher for newborns in the supplemented group, with a mean difference of 107.6 g (95% confidence interval [CI]: 59.9–155.3 g) and 0.3 cm (95%CI: 0.10–0.41 cm), respectively, and that the levels of 25 (OH) D were significantly higher in the supplemented group, but the incidence of pre-eclampsia, GDM, low gestational age, low birthweight, premature birth, and cesarean section were not influenced by vitamin D supplementation, needing the elaboration of studies with larger populations to reach a definitive conclusion. 16 17

A case-control study that included 4,718 women, designed to assess maternal blood serum concentrations of 25 (OH) D and its association with GDM and other pregnancy outcomes, found that concentrations of 25 (OH) D were significantly lower in pregnant women with GDM compared with the control group. After adjusting for confounding factors, women with low concentrations of 25 (OH) D had a significantly increased risk of GDM and of some adverse pregnancy outcomes (anemia, macrosomia, abnormal amniotic fluid, and miscarriage or stillbirth). A threshold of 25 (OH) D of 50.0 nmol was also observed for the development of GDM. 18

A recent systematic review with meta-analysis from randomized clinical trials, carried out by the authors of this same review sought to assess the effectiveness of vitamin D supplements used alone and in combination with calcium and vitamin supplements in pregnant women with GDM through the analysis of relevant maternal and neonatal parameters. The authors concluded that vitamin D supplementation in pregnant women with GDM may contribute to a decrease in fasting blood glucose (MD: −18.64 [−24.31–12.97]; *p* < 0.00001), homeostasis model assessment for β *-* cell function (HOMA- β) and homeostasis model assessment for insulin resistance (HOMA-IR) (MD: −1.59 [−c2.20; −0.98); *p* < 0.00001), serum calcium (MD: 0.60 [0.29–0.60]; *p* = 0.0002), complications of the newborn, such as the occurrence of hyperbilirubinemia and polyhydramnios (MD: 0.34 [0.20-0.58]; *p* < 0.0001), need for maternal hospitalization (MD: 0.13 [0.0–,0.98], *p* = 0.05) and newborn hospitalization (MD: 0.40 [0.23–0.68]; *p* = 0.0008), and increased concentration of 25 (OH) D (MD: 16.63 [11.46–21.80); *p* < 0.00001). However, these results should be interpreted with caution, as the quality of the evidence obtained through the Grading of Recommendations, Assessment, Development and Evaluations (GRADE) tool was very low and the studies included in the review present a risk of high bias and small sampling. The effect of vitamin D when combined with other vitamins and minerals should be clarified. Therefore, other randomized clinical trials with placebo should be designed to highlight the possible benefits of supplementation for pregnant women with GDM. 19

A cohort study that aimed to determine vitamin D levels after pregnancies affected by GDM and to verify its association with β cell function, insulin resistance or diabetes diagnosis in the future. This study identified that low levels of 25 (OH) D3 were common up to 2 years after the occurrence of GDM. The study suggests that vitamin D deficiency/insufficiency appears to be associated with β cell dysfunction and insulin resistance. However, no association has been reported between vitamin D levels and the development of type 2 diabetes, and further studies are needed in the future for clarification. 20

Women who have developed GDM often have postpartum glucose intolerance, as well as increased insulin resistance after delivery, compared with normoglycemic women. Increased insulin resistance is likely to increase the risk of metabolic syndrome and of type 2 diabetes in subsequent years. Thus, postpartum vitamin D supplementation in women with GDM can be suggested as an intervention to protect against β cell dysfunction, insulin resistance, and the diagnosis of type 2 diabetes in the future. In view of the above, the present review aimed to evaluate the effects of vitamin D supplementation in the postpartum period of pregnant women with previous GDM.

## Methods

### Protocol and Registration

This is a bibliographic study, a systematic review with meta-analysis performed according to the Cochrane methodology. 21 The present systematic review had its protocol published in the PROSPERO (International prospective register of systematic reviews) database under the CRD42018110729 22 register and followed the rules of the Preferred Reporting Items for Systematic Reviews and Meta-Analyses (PRISMA) checklist. 23

### Eligibility Criteria

Randomized clinical trials (RCTs) that evaluated the effects of vitamin D supplementation in the postpartum period of pregnant women with previous GDM were selected, with 3 months of follow-up and with evaluation of the results in the postpartum period, in which the patients were randomly distributed into two groups: intervention group and control group, following the PICO methodology described below. Population: pregnant women diagnosed with GDM who received vitamin D supplementation in the prenatal or postpartum period. Intervention: effect of vitamin D supplementation in the postpartum period. Comparator: no supplementation and/or placebo. Outcomes: serum vitamin D concentration (25-hydroxyvitamin D in nmol/L), fasting glucose, glycated hemoglobin, serum calcium concentration, HOMA-IR, quantitative insulin sensitivity check index (QUICKI), parathyroid hormone (PTH), and body mass index (BMI).

### Inclusion and Exclusion Criteria

The present study evaluated vitamin D supplementation in the postpartum period, used alone and in combination with calcium and vitamin supplements, on the maternal results of pregnant women with previous GDM. Randomized clinical trials of pregnant women with GDM of any chronological, gestational age and parity, with no history of previous disease who received vitamin D supplementation in the prenatal and/or the postpartum period and were evaluated in the postpartum period were included. The intervention of interest was: vitamin D isolated in the postpartum versus no prenatal and/or postpartum placebo treatment or administration. Intervention and control can be administered by any means. The exclusion criteria were: evaluation of the pregnant woman in the prenatal period, < 3 months of follow-up, and nonrandomization between the groups.

### Search Strategy

The following electronic databases were consulted: the National Center for Biotechnology Information (NCBI/PubMed) (1966–July 2019), Embase (1980–March 2019), Cochrane Library (1972–July 2019), Latin American Literature and Caribbean Health Sciences (LILACS) (1982–July 2019), and the Virtual Health Library (VHL) website. Information on ongoing clinical trials was retrieved through the clinical trials website ( http://clinicaltrials.gov ) of the National Health Institute and through the Brazilian Registry of Clinical Trials (ReBEC, in the Portuguese acronym) ( http://www.ensaiosclinicos.gov.br/ ). The basic search strategy was developed for PubMed and was modified as needed for other databases ( **Appendix 1** ). The health descriptors available in Health Sciences Descriptors (DECs) and Medical Subject Heading (MeSH) were used. The descriptors used included *gestational* *diabetes* , *postpartum period* , *vitamin D* , and *cholecalciferol* . There was no language restriction, but only human studies were selected. References of selected articles, including relevant review articles, were reviewed to identify all relevant studies. Manual search for references of clinical trials in relevant journals was performed.

**Chart 1:** Characteristics of included studies in a systematic review and meta-analysis of randomized controlled trials to evaluate the effects of vitamin D supplementation in the postpartum period of pregnant women with previous gestational diabetes mellitus

First author, year published	Study location	Source of funding	No. of participants	Age (years old), percentiles	Treatment duration	Inclusion criteria	Exclusion criteria	Treatment group	Primary endpoint
Valizadeh et al. (2016) 27	Iran	Zanjan University of Medical Sciences and Farir-Teb Company	84	G1: 32.0 (5.5) G2: 32.4 (4.7)	12 weeks after delivery	Pregnant women diagnosed with GDM * – **, maternal age > 16 years, single pregnancy and gestational age between 12 and 32 weeks	Women with type 1 or 2 diabetes known before pregnancy, history of hypertension or thyroid disorders. Women who used assisted reproduction techniques or those with a history of consuming high doses of vitamin D during the previous three months	G1: 200.000 IU of vitamin D3 in the first two days and then 50.000 IU per week, up to 700.000 IU in total. G2: received nothing	Fasting plasma glucose, 2-hour post 75 g glucose load plasma glucose (2-hPLG), fasting serum insulin, homeostasis model assess- ment of insulin resistance (HOMA-IR), HbA1C, and serum 25OHD at 6 - 12 weeks after delivery.
Yeow et al. (2015) 28	Malaysia	Penang Medical College and Medical Research Grant from Ministry of Health, Malaysia	26 G1:13 G2:13	G1:36 (32, 38) G2: 35 (30, 40	6 months after delivery	Women diagnosed with GDM in the last pregnancy, who were between 6 to 48 months after delivery	Women with diabetes, pregnancy, breastfeeding, intolerance to vitamin D supplementation, alcohol dependence, drug use, chronic kidney or liver failure, hypercalcemia, hypocalcemia or concomitant use of calcium supplementation, treatment for tuberculosis or anti-epileptic medications.	G1: 4000 IU of vitamin D3 (cholecalciferol) per day (four capsules of 1000 IU each) for six months G2: four corresponding placebo capsules per day for six months	Serum vitamin D level (nmol/L) Intact parathyroid hormone (pg/mL) HbA1C level and glucose metabolism during OGTT Fasting glucose (mmol/L) 30-minute glucose (mmol/L) 2-hour glucose (mmol/L) AUCglucose (mmol/L) ΔAUCglucose (mmol/L) Insulin secretion Fasting insulin (pmol/L) Fasting C-peptide (ng/mL) Insulin sensitivity OGIS (ml/ min/ m2) BIGTT-S (10–5*(min*pmol/L)-1) Disposition index (OGIS* ratio of total AUCinsulin over AUCglucose)
Hosseinzadeh-Shamsi-Anar et al. (2012) 29	Iran	NI	45 G1:24 G2:21	G1: 30.7 ± 6.2 G2: 29.5 ± 4	3 months after delivery	Pregnant women diagnosed with GDM, absence of thyroid, kidney and liver diseases, as well as malabsorption	Change in routine treatment and intake of vitamin D, Ca and multivitamin supplements.	G1: 300.000 IU of vitamin D3 administered intramuscularly in the diagnosis of GDM G2: Did not receive placebo	glycosylated hemoglobin A1C (HBA1C), serum 25-OH-D, parathyroid hormone (PTH), serum calcium, phosphorus, BMI (kg/m2) and HBA1C (%)
Mozaffari-Khosravi et al. (2012) 30	Iran	NI	45 G1:24 G2:21	G1: 30.7 ± 6.2 G2: 29.5 ± 4	3 months after delivery	Women diagnosed with GDM ** in the last pregnancy, absence of thyroid disease, kidney and liver diseases and absence of malabsorption	NI	G1: 300,000 IU of vitamin D3 administered intramuscularly after delivery G2: Did not receive placebo	HbA1c, serum 25-hydroxyvitamin D3, fasting insulin and blood glucose, C-peptide, homeostasis model assessment insulin resistance index (HOMA-IR),

Abbreviations: G1, intervention group; G2, control group; GDM, gestational diabetes mellitus; OGIS, oral glucose insulin sensitivity; OGTT, Oral Glucose Tolerance Test; NI, not informed.

* ADA: American Diabetes Association guidelines

** Carpenter and Coustan criteria

### Selection of Studies and Data Extraction

For the present review, two researchers independently reviewed the eligibility of the titles and summaries. Disagreements regarding the selection of articles were resolved either by consensus or by discussion with a third investigator. The study selection flowchart was created in accordance with the PRISMA guidelines. 23

Two researchers independently extracted the relevant data (participants, specific vitamin D intervention, and outcome characteristics) from each full-text article using a standardized form based on the Cochrane Handbook 21 with the following information: study characteristics (design, randomization method); participants; interventions; clinical outcomes (types of outcomes measured, that is, dichotomous or continuous; adverse effects). The selection was compared for accuracy, and any discrepancies were resolved either by consensus or by discussion with another investigator.

### Bias Risk Assessment

Two investigators independently assessed the risk of bias for each eligible RCT. Discrepancies were resolved either by consensus or by discussion with another investigator. The Cochrane Collaboration tool was used to assess the risk of bias in RCTs. 24 Thus, the items evaluated were: generation of the allocation sequence (selection bias); hiding the allocation sequence (bias selection); blinding (detection and performance bias); blinding of participants and staff for evaluation of results; incomplete result data (attrition bias); selective reporting result (information bias). For each RCT, the items were described and presented as low risk of bias, risk of uncertain bias or high risk of bias according to the classification obtained.

### Data Analysis

For the analysis, fixed and random effects models (when necessary) and the Mantel-Haenszel method were used. Associations were reported as relative risks (RR) and their 95% confidence intervals (CIs). Standard deviation (SD) was calculated when the interquartile range (IQR) was available. Heterogeneity was tested with the Cochrane χ ^2^ test, and the degree of heterogeneity was quantified with the I2 statistic and its 95%CI. An I2 value between 30 and 60% was described as moderate heterogeneity. Publication bias was assessed with the funnel plots and formally tested with the Egger test. For the variability in results between studies, the I2 statistic and the p *-* value obtained from the chi-squared Cochrane test were used. Review Manager (RevMan) software was used for all analyzes (version 5.3; Nordic Cochrane Center, Cochrane). 25 Conversion factor: 1 mmol/L = 18.018 mg/dL; pg/ml was converted to pmol/l when necessary.

### Assessment of the Quality of Evidence

The evaluation of the quality of the evidence was made with the GRADE tool 26 for the outcomes serum vitamin D concentration (25-hydroxyvitamin D in nmol / L), fasting glycemia, glycated hemoglobin, serum concentration of calcium, HOMA-IR, QUICKI, PTH and BMI.

## Results

### Selected Articles

**Fig. 1:**
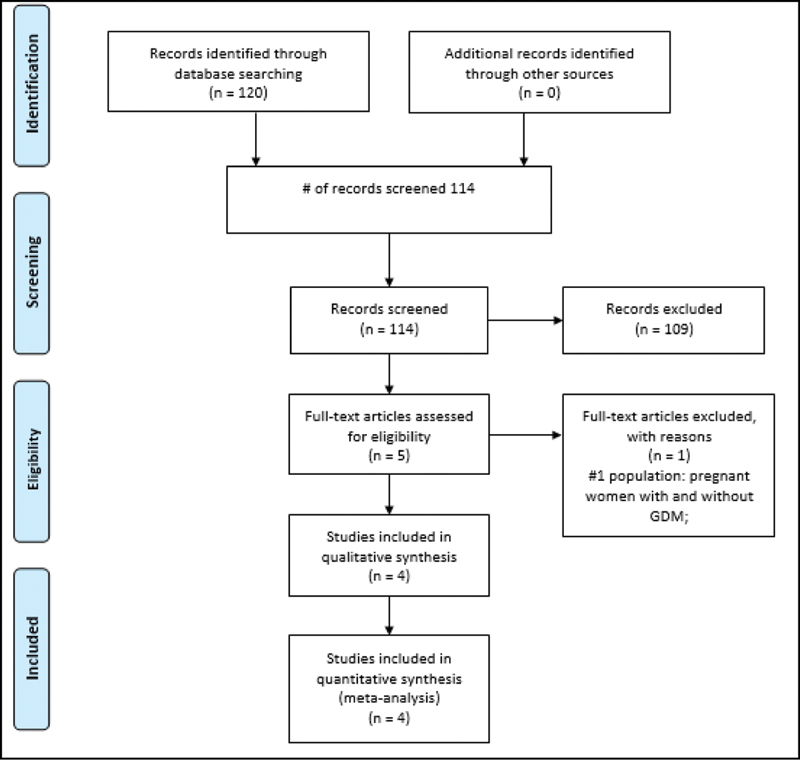
Flowchart for identifying eligible studies.

After searching the electronic health databases, 120 references were identified. Seven articles were potentially eligible for inclusion in the present review and, therefore, were read in full. After reading and critical analysis, four articles were selected for qualitative and quantitative analysis (meta-analysis). The gray literature did not report any findings according to the eligibility criteria ( Fig. 1 ).

After being read in full, four studies met the inclusion criteria and were included in the present systematic review. 27 28 29 30 One study was excluded because its population included women without a previous diagnosis of GDM. 31

### Description of the Studies

The 4 included RCTs totaled 200 pregnant women diagnosed with previous GDM who received vitamin D supplementation in the prenatal or postpartum period. 27 28 29 30 In two articles, supplementation occurred during prenatal care, 27 29 and, in the other two, supplementation was administered in the postpartum period, 28 30 but all analyzes were performed in the postpartum period. In only one study there placebo was administered in the control group, while in the intervention group 4000 IU of vitamin D3 (cholecalciferol) per day (4 capsules of 1000 IU each) were administered for 6 months; in the control group, placebo was administered similarly to Vitamin D3 in the intervention group. 28 Another study that administered vitamin D supplementation (25-hydroxyvitamin D in nmol/L) orally, through the administration of capsules, had an intervention of 200,000 IU of vitamin D3 in the first 2 days after randomization, followed by 50,000 IU per week thereafter, up to a total of 700,000 IU. Women randomized at ≥ 28 weeks of gestation received 100,000 IU weekly. 27 In the other 2 studies, the control group also did not receive a placebo while individuals in the intervention group received a single-dose intramuscular injection containing 300,000 IU of vitamin D3. 29 30 Chart 1 describes the characteristics of the studies included in the analysis.

### Risk of Bias

**Fig. 2:**
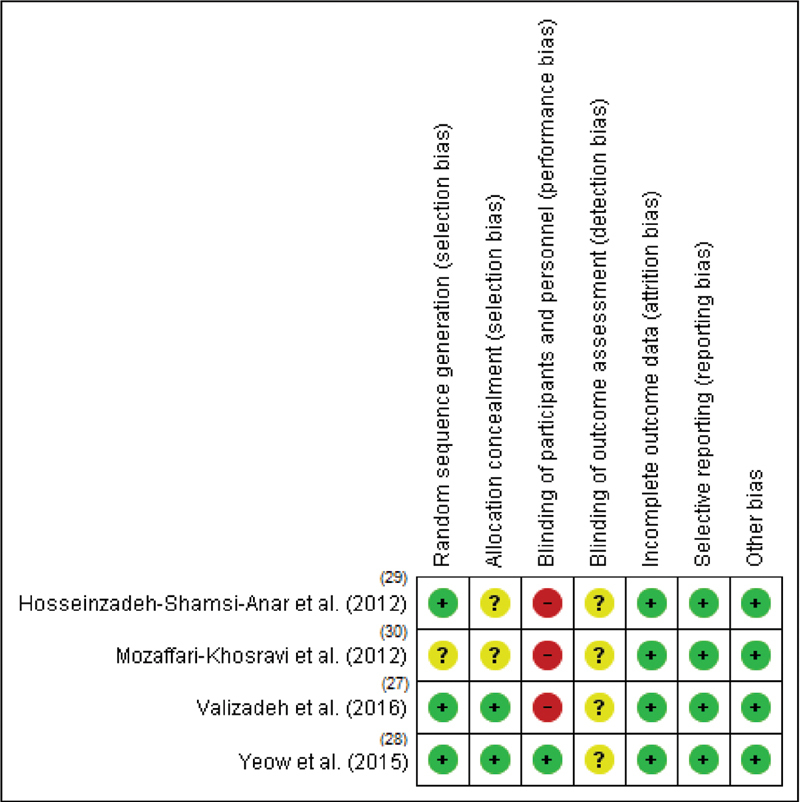
Assessment of bias risk of randomized clinical trials included.

The risk of bias assessment is summarized in Fig. 2 . Regarding the randomization process, three 27 28 29 studies were considered as of low risk, since one reported the use of computer software to generate the random sequence for randomization 28 and two reported the use of a table of random numbers. 27 29 One study was classified as of uncertain risk, as it did not report the randomization process. 30

In the allocation concealment, two 29 30 studies were considered uncertain because they did not report the process and two 27 28 were of low risk, since one reported that the concealment was preserved by the existence of opaque envelopes numbered sequentially 27 and the other study was considered low risk because reported that the drugs were number in a different way sequential with identical appearance. 28

Regarding the blinding of participants and professionals, three 27 29 30 studies included in the present review were considered as of high risk, since no placebo was administered in the control group. One study 28 was considered as of low risk because placebo was administered at the same dose as the intervention and there is evidence that randomization and allocation were hidden from investigators and subjects until the study was completed.

In the blinding outcome evaluation, the four 27 28 29 30 studies were considered uncertain because they did not report this information.

Regarding incomplete outcomes, the four 27 28 29 30 studies were considered as of low risk, with one 28 reporting performing analysis by intention to treat and three 27 29 30 describing their losses and reasons, with balanced data losses between groups, with similar reasons for data loss between groups.

For reporting a selective outcome, the four 27 28 29 30 studies were classified as of low risk of bias, with two 28 29 studies with records and results compatible with those initially proposed and two 27 30 studies that, even without records, reported outcomes compatible with the intervention. Regarding other sources of bias, they were not identified in any study, with the four studies 27 28 29 30 being classified as of low risk of bias.

### Meta-analysis

**Fig. 3:**
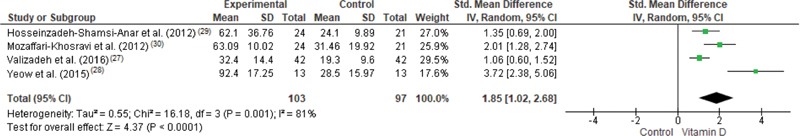
Meta-analysis of serum vitamin D concentration.

Serum vitamin D concentration (25-hydroxyvitamin D in nmol/L), fasting glycemic parameters, glycated hemoglobin, serum calcium concentration, HOMA-IR, QUICKI, PTH, and BMI were considered primary outcomes. The four 27 28 29 30 studies included in the present review analyzed the serum concentration of vitamin D after follow-up, where the meta-analysis identified that in the group that received vitamin D in the prenatal and/or in the postpartum period, but were analyzed later, there was a significant increase in the serum concentration of vitamin D in the intervention group (RR: 1.85; 95%CI: 1.02–2.68), but with high heterogeneity (I2 = 81%) between the studies ( Fig. 3 ).

**Fig. 4:**
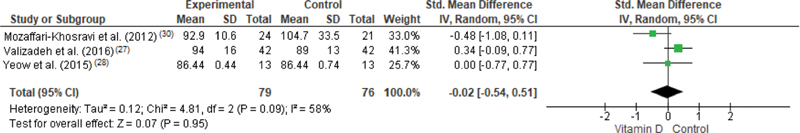
Meta-analysis of fasting blood glucose.

Fasting blood glucose levels were reported in three 27 28 30 articles and demonstrated no difference between the control and intervention groups that received vitamin D supplementation in the postpartum period of pregnant women with previous GDM (RR: −0.02; 95%CI: −0.54–0.51), showing medium heterogeneity (I2 = 58%) between the studies ( Fig. 4 ).

**Fig. 5:**
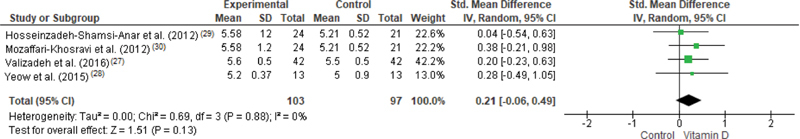
Meta-analysis of glycated hemoglobin.

The outcome was glycated hemoglobin across all 27 28 29 30 studies and showed no significant difference between groups (RR: 0.21; 95%CI: −0.06–0.49) with null heterogeneity (I2 = 0%) between the studies ( Fig. 5 ).

**Fig. 6:**
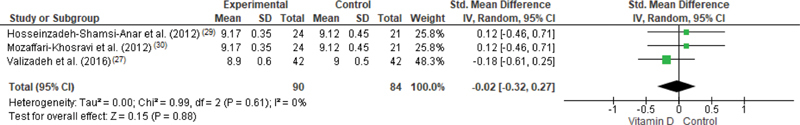
Meta-analysis serum calcium concentration.

For serum calcium concentration, there was an analysis in three studies, 27 29 30 in which the meta-analysis showed no statistically significant difference between the groups (RR: - 0.02; 95%CI: - 0.32–0.27) with null heterogeneity (I2 = 0%) between the studies ( Fig. 6 ).

**Fig. 7:**
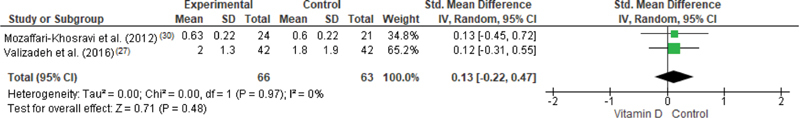
Meta-analysis HOMA-IR.

The HOMA-IR outcome was reported in 2 30 studies and showed no significant difference between groups (RR: 0.13; 95%CI: - 0.22–0.47) with null heterogeneity (I2 = 0%) between the studies ( Fig. 7 ).

**Fig. 8:**
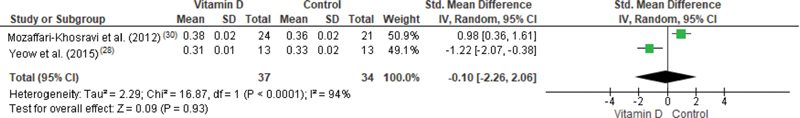
Meta-analysis QUICKI.

The QUICKI outcome was also reported in 2 30 studies and showed no significant difference between groups (RR: - 0.10; 95%CI: - 2.26–2.06) with high heterogeneity (I2 = 94%) between the studies ( Fig. 8 ).

**Fig. 9:**
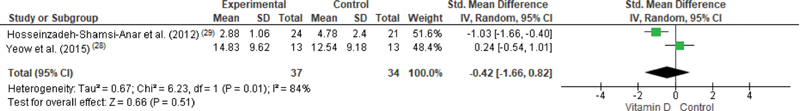
Meta-analysis of parathyroid hormone (PTH).

Parathyroid hormone was analyzed in 2 29 studies and showed no significant difference between groups (RR: - 0.42; 95%CI: - 1.66–0.82) with high heterogeneity (I2 = 84%) between the studies ( Fig. 9 ).

**Fig. 10:**
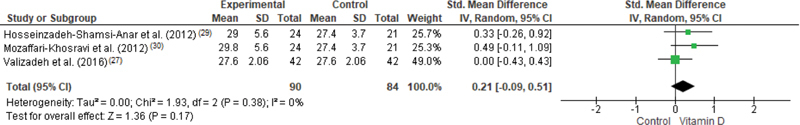
Meta-analysis of body mass index (BMI).

Body mass index was analyzed in 3 29 30 studies and did not show a significant difference between the groups (RR: 0.21; 95%CI: - 0.09–0.51) with null heterogeneity (I2 = 0%) between the studies ( Fig. 10 ).

### Evaluation of Quality of Evidence According to the GRADE framework

The evaluation of the quality of the evidence was performed for the outcomes serum vitamin D concentration (25-hydroxyvitamin D in nmol /L), fasting blood glucose, glycated hemoglobin, HOMA-IR, QUICKI, PTH, all of which are classified as very low quality of evidence. As < 10 RCTs were included in the present review, it was not possible to analyze the presence of publication bias ( Table 1 ).

**Table 1:** Summary of findings based on the GRADE framework

Outcomes	Patients (n)	Risk of bias	Inconsistency	Indirectness	Imprecision	Publication bias	Quality of evidence
Serum vitamin D concentration							
	200 (4 RCTs)	Serious(−1)*	Very serious ^a^ (-2)*	Not serious	Serious ^b^ (-1)*	Probably not	ⴲ◯◯◯very low
Fasting blood glucose							
	132 (3 RCTs)	Serious(-1)*	Serious ^a^ (-1)	Not serious	Serious ^b^ (-1)*	Probably not	ⴲ◯◯◯very low
Glycated hemoglobin							
	200 (4RCTs)	Serious (-1)*	Not serious	Not serious	Very serious ^b^ (-2)*	Probably not	ⴲ◯◯◯very low
HOMA-IR							
	129 (2 RCTs)	Serious (-1)*	Not serious	Not serious	Very serious (-2)*	Probably not	ⴲ◯◯◯very low
QUICKIE							
	68 (2 ECR's)	Serious (-1)*	Very serious ^a^ (-2)*	Not serious	Not serious	Probably not	ⴲ◯◯◯very low
PTH							
	71 (2 ECR's)	Grave (-1)*	Very serious ^a^ (-2)*	Not serious	Not serious	Probably not	ⴲ◯◯◯very low

a. High heterogeneity between studies, b. Amplitude in the 95% confidence interval

Note: To determine a GRADE quality of the evidence, the GRADE approach begins by assigning findings to one of the two initial levels of quality, depending on the study design. Randomized trials are of high quality, while observational studies are of low quality. The evidence can be considered at four levels: high, moderate, low, and very Low. Studies can be updated or downgraded based on certain factors:

a) Risk of bias (−1 if serious risk of bias, −2 if very serious risk of bias).

b) Inconsistency or heterogeneity of evidence (−1 if serious inconsistency, −2 if very serious inconsistency)

c) Indirectness of evidence (−1 if serious, −2 if very serious)

d) Imprecision of results (−1 if wide confidence interval, −2 if very wide confidence interval)

e) Publication bias (−1 if likely, −2 if very likely) *Small events and a large confidence interval. Low quality of evidence: the authors do not trust the estimate of the effect and the actual value may differ substantially from this.

## Discussion

In the literature, the present article is the first systematic review evaluating the effects of vitamin D supplementation in the postpartum period of pregnant women with previous GDM.

Previously, a systematic review with meta-analysis performed by at least one research group aimed to assess whether vitamin D supplementation administered to pregnant women with GDM would improve maternal and neonatal outcomes and found no evidence of moderate or high quality indicating that vitamin D supplementation, when compared with placebo, improves glucose metabolism or adverse maternal and neonatal outcomes related to GDM. 19

Our findings indicate that there is no difference in the postpartum period in women diagnosed with previous GDM who received vitamin D supplementation in the prenatal and/or in the postpartum period, showing only that there was a significant increase in the concentration of vitamin D (RR: 1.85; 95%CI: 1.02–2.68). This increase in the concentration of vitamin D should be interpreted with caution, since the assessment of the quality of the evidence was very low. For the other outcomes analyzed, there was no significance between the intervention and control groups.

The present systematic review has limitations, the main one being related to the small number of clinical trials and of women included in the analysis. The option to include only RCTs can also be a limiting factor for the analyzes; however, the choice was based on the search for studies that reported the best design to obtain the best available evidence.

The small number of clinical trials included resulted in a small population analyzed, and this factor contributed to the extensive CIs between the studies.

The methodological quality of RCTs can also be considered a limiting factor, since the selected studies have questionable methodological biases. The fact that two studies did not administer a placebo in the control group made it impossible to blind participants and researchers.

## Conclusion

No moderate or high-quality evidence was found in the included RCTs that prove that there are favorable effects of vitamin D supplementation in the postpartum period of pregnant women with previous GDM. Thus, there is no evidence to suggest that vitamin D supplementation may be a protective factor against β cell dysfunction, insulin resistance and the diagnosis of type 2 diabetes in the future. The development of well-designed RCTs with the inclusion of large populations is recommended, as well as the use of placebos in the control group to verify the efficacy and safety of vitamin D supplementation in the postpartum period of pregnant women with previous GDM with the aim of to verify whether supplementation can beneficially assist in maintaining β cell function, in reducing insulin resistance and, in the future, reducing the incidence of type 2 diabetes.

## Notes

Appendix 1 #1 "Diabetes, Gestational"[Mesh] OR (Diabetes, Pregnancy-Induced) OR (Diabetes, Pregnancy Induced) OR (Pregnancy-Induced Diabetes) OR (Gestational Diabetes) OR (Diabetes Mellitus, Gestational) OR (Gestational Diabetes Mellitus) OR (GDM) #2 "Pregnancy in Diabetics"[Mesh] OR Pregnancy in Diabetic OR Pregnancy in Diabetes OR Pregnancy in Diabete #3 "Vitamin D"[Mesh] OR (D04.808.812.768) OR (D014807) OR (1406-16-2) OR (Vitamin D3) OR (25(OH)D) OR (25-hydroxyvitamin D) #4 "Cholecalciferol"[Mesh] OR (Calciol) OR ((3 beta,5Z,7E)-9,10-Secocholesta-5,7,10(19)-trien-3-ol) OR (Vitamin D 3) OR (Vitamin D3) OR (Cholecalciferols) OR (D04.808.247.222.159) OR (D04.808.247.808.146) OR (D04.808.812.768.196) OR (D10.570.938.146) OR CD002762) OR (1C6V77QF41) #5 "Postpartum Period"[Mesh] OR Period, Postpartum OR Postpartum OR Postpartum Women OR Women, Postpartum OR Puerperium (#1 OR #2) AND (#3 OR #4) AND #5
